# Health managers’ views on the status of national and decentralized health systems for child and adolescent mental health in Uganda: a qualitative study

**DOI:** 10.1186/s13034-015-0086-z

**Published:** 2015-12-08

**Authors:** Angela Akol, Ingunn Marie Stadskleiv Engebretsen, Vilde Skylstad, Joyce Nalugya, Grace Ndeezi, James Tumwine

**Affiliations:** Faculty of Medicine and Dentistry, Center for International Health, University of Bergen, Bergen, Norway; Department of Psychiatry, Mulago National Referral Hospital Kampala, Kampala, Uganda; Department of Pediatrics and Child Health, Makerere University College of Health Sciences Kampala, Kampala, Uganda

**Keywords:** Child Adolescent Mental Health, Health systems (decentralized), Qualitative research, Sub-Saharan country, Uganda, Africa

## Abstract

**Background:**

Robust health systems are required for the promotion of child and adolescent mental health (CAMH). In low and middle income countries such as Uganda neuropsychiatric illness in childhood and adolescence represent 15–30 % of all loss in disability-adjusted life years. In spite of this burden, service systems in these countries are weak. The objective of our assessment was to explore strengths and weaknesses of CAMH systems at national and district level in Uganda from a management perspective.

**Methods:**

Seven key informant interviews were conducted during July to October 2014 in Kampala and Mbale district, Eastern Uganda representing the national and district level, respectively. The key informants selected were all public officials responsible for supervision of CAMH services at the two levels. The interview guide included the following CAMH domains based on the WHO Assessment Instrument for Mental Health Systems (WHO-AIMS): policy and legislation, financing, service delivery, health workforce, medicines and health information management. Inductive thematic analysis was applied in which the text in data transcripts was reduced to thematic codes. Patterns were then identified in the relations among the codes.

**Results:**

Eleven themes emerged from the six domains of enquiry in the WHO-AIMS. A CAMH policy has been drafted to complement the national mental health policy, however district managers did not know about it. All managers at the district level cited inadequate national mental health policies. The existing laws were considered sufficient for the promotion of CAMH, however CAMH financing and services were noted by all as inadequate. CAMH services were noted to be absent at lower health centers and lacked integration with other health sector services. Insufficient CAMH workforce was widely reported, and was noted to affect medicines availability. Lastly, unlike national level managers, lower level managers considered the health management information system as being insufficient for service planning.

**Conclusion:**

Managers at national and district level agree that most components of the CAMH system in Uganda are weak; but perceptions about CAMH policy and health information systems were divergent.

## Background

Child and adolescent mental health (CAMH) is essential for optimal social and psychological well-being and development. Early detection and management of CAMH disorders reduces the likelihood of long term ill health and minimizes stress on individuals, families, communities and health systems [1]. Robust health service systems are required for the promotion of CAMH [2]. Up to 20 % of children and adolescents globally suffer from a debilitating mental illness and up to 50 % of adult mental illness begins in adolescence [3]. In low and middle income countries such as Uganda, the associated disability-adjusted life years (DALYs) from neuropsychiatric illness in childhood and adolescence represent 15–30 % of all DALYs lost [4, 5]. In spite of this burden, service systems in countries with the largest proportion of children and adolescents are weak [6].

Three out of four objectives in the World Health Organization’s (WHO) mental health plan of action focus on improving the mental health care system [7]. Under this plan, the WHO aspires to increase service coverage for mental health disorders in all countries. Because provision of CAMH services depends on the availability of necessary policies, funding, integrated services, preventive and therapeutic services underlined by evaluation and research, [8] the expansion of CAMH services necessitates strengthening of all these areas.

In Uganda, a mental health policy was developed in 2000. However the policy did not address CAMH until it was complemented and improved by child and adolescent mental health policy guidelines developed in 2014. Nevertheless, important areas not addressed by policy are mental health financing; service quality improvement; the role of psychologists and social workers; and conflict and mental health [9]. Mental health policy is governed under the overarching second national health policy (2010) and its attendant health sector strategic and investment plan (HSSIP III). The HSSIP III makes a reference to mental health as a government priority within the non-communicable diseases cluster of the minimum health care package. However CAMH is not mentioned [10].

The policies are supported under several legislative instruments which protect the needs and rights of children. Specifically, Article 34 of the 1995 Constitution of the Republic of Uganda provides for the following rights and protection of children: the right to know and be cared for by their parents or other people; the right to basic education; the right not to be denied medical treatment or any social or economic benefits; protection from all exploitation including employment and work that is harmful to their health or education; and the right of child offenders not to be detained with adults. The Children Act 1996—chapter 59 laws of Uganda—puts into effect the constitutional provisions on children while the Mental Health Treatment Act (1964) provides for custodial care of mentally ill persons, but according to Kigozi et al. [9] “is not in accordance with contemporary international human rights standards regarding mental health care” [9, page 3].

Uganda’s health system is divided into national and district-based levels. At the national level are the National Referral Hospitals, Regional Referral Hospitals (RRH) and semi-autonomous institutions like the Uganda Blood Transfusion Services and the Uganda National Health Research Organization [11]. The lowest rung of the district-based health system consists of Village Health Teams (VHTs), who are community health volunteers delivering predominantly health education and preventive services in communities. The next level is Health Center II (HC II) which is an outpatient service run by a nurse. Next in level is health Center III (HC III) which provides in addition to HC II services, in patient, simple diagnostic and maternal health services. It is managed by a clinical officer who does not have a medical degree. Above a HC III is the Health Center IV, run by a medical doctor and providing surgical services in addition to all the services provided at HC III. The HC IV is also referred to as a health sub district (HSD) and has supervisory responsibility over HC IIIs and HC IIs in its catchment area. Thus, the medical doctor who runs the HC IV is also called the HSD manager [10].

The most recent information on the organization of mental health services in Uganda comes from a 2005 survey based on the World Health Organization’s assessment instrument for mental health systems (WHO-AIMS). This survey reports that mental health services in Uganda consist of 28 outpatient and 27 in-patient units in the country, at the psychiatric units of all hospitals outside the national mental health referral hospital. While 15 % of the 382 mental hospital beds in these units are reserved for children and adolescents, none of the out-patient clinics is specialised for CAMH. One 500-bed mental hospital with a forensic in-patient unit serves as a national referral hospital. There are 1.28 psychiatric beds per 100,000 Ugandans, below the global and high-income country averages of 6.5 and 41.8, respectively [12]. All services are coordinated by one principal medical officer at the Ministry of Health. Mental health services receive approximately 1 % of Uganda’s health sector budget [9, 13, 14], compared to a global median of 2.8 and 5.1 % in high income countries [15].

The burden of CAMH disorders in Uganda has not been accurately estimated. Nalugya et al. [16] estimated the burden of depression among Ugandan secondary school students in one district at 21 %; and Okello et al. [17] estimated that approximately 44 % of war-affected adolescents in another district suffered from one or more CAMH disorder. A discussion of CAMH disorders in Uganda is limited by a paucity of epidemiologic data.

To our knowledge, besides quantitative studies undertaken in 2001 and 2005 as part of global WHO-led surveys, no qualitative assessment of CAMH systems has been conducted in Uganda. Thus, the objective of our assessment was to explore strengths and weaknesses of CAMH systems at the national and district level in Uganda from a management perspective, in order to inform the implementation of national mental health policy. We present findings from the health system at national level and one decentralized health system, in Mbale district, eastern Uganda. A management perspective is selected because managers are best placed to provide users’ understanding of system-wide operations, including analysis of non-clinical aspects like finances, supplies and personnel. A managers’ viewpoint is also considered necessary to complement previous work, which has only been done from an external evaluator’s viewpoint as part of global WHO mental health surveys. Two such surveys using quantitative methods were conducted in 2001 and 2005.

## Methods

### Study site

Uganda is situated in East Africa with a population of 34.9 million [18]. Mbale district in eastern Uganda is the site for the ongoing study “SeeTheChild—Mental Child Health in Uganda” which aims to characterize the most common psychiatric conditions among children and assess the related health system aspects. Mbale also provides an informative case study because it personifies all levels of decentralized health services from (RRH) to village health workers. Mental health in-and out-patient services in Mbale district are provided at the psychiatric unit of the Mbale RRH, which does not have a psychiatrist on staff. The leader of the district health system is the District Health Officer (DHO).

### Study design

Seven key informant interviews were held with all public officials responsible for management and supervision of CAMH services at national (Kampala) and district level (Mbale). All seven eligible managers were interviewed. Interviews were conducted during July to October 2014 in Kampala and Mbale district, Eastern Uganda. Key informant interviews are judged to be an appropriate methodology because they delve into the subject in question from the perspective of individuals who have knowledge of the subject by virtue of their natural position [19]. Guest and colleagues noted that 6–12 interviews are sufficient to deliver data saturation in a homogenous, purposively selected sample, with enquiry into a concise subject [20]. As this corresponds to our research, seven interviews were considered sufficient for our purpose.

Four of the key informants were female and six were medical doctors; two with specialization as psychiatrists. Four of the informants were district based managers and three were based at the national level. The management experience of the managers ranged from 3 months to 20 years, with a median duration of 4 years. All officials who were approached for interviews accepted to participate.

### Data collection

The interviews were conducted with a semi-structured interview guide divided into the following CAMH-related domains of enquiry:

- Policy and legislation
- Financing
- Service delivery
- Health workforce
- Medicines
- Health information management and research

The interview schedule was developed by the principal investigator (PI), based on domains in the World Health Organization’s Assessment Instrument for Mental Health Systems (WHO AIMS) Version 2.2 [21]. Using this instrument as a guide, open ended questions were developed around assessment items listed, adapting to context and cadre of manager. For example, under Domain 1 of the WHO-AIMS tool, open ended questions were crafted to elicit managers’ views on the items comprising policy and legislative framework as listed by WHO-AIMS. Items that were not appropriate for the health managers were excluded from the interview guide, for example questions on national monitoring of human rights.

The interviews were conducted in English by the PI (AA) and recorded verbatim. All the interviews took place in the officials’ offices, except for one interview which was conducted on-line as a voice interview using the application Skype due to the official’s absence from their duty station. The interviews lasted 25–40 min, were audio-taped and notes were taken.

### Analysis

The recorded interviews were transcribed, followed by inductive thematic analysis applied to all the data, based on methods described by Guest et al. [22] and Vaismoradi et al. [23]. A code-sheet was developed by the principal investigator with all the relevant themes. The text contained in the transcripts was reduced to thematic codes and within those themes, content codes were developed. Patterns were then identified in the relations among the codes. The PI lead the analysis and the raw material was co-read by one of the co-authors (IE).

This assessment was conducted within the research project “SeeTheChild—Mental Child Health Study in Uganda” (Research Council of Norway (http://www.rcn.no) project number: 220887), an ancillary study to the follow-up study ‘The PROMISE Saving Brains study in Uganda and Burkina Faso’ (ClinicalTrials.gov #NCT01882335). The assessment commenced after ethical approval was received from the Research ethics committee, School of Medicine, Makerere University reference number 2012-177. Written informed consent was obtained from the participants.

## Results

Results are presented according to domains in the WHO-AIMS version 2.2. Eleven themes emerged under each of the six domains of enquiry (Table 1). Adequacy of each of the domains became apparent as an overarching theme, and participants discussed the competence or insufficiency of the different domains. Illustrative quotes from the interviews are provided for each theme.

**Table 1:** Themes that emerged during the analysis of the data

Domain	Main themes	Content code	Tally of responses by level of manager			Total
			National	District	Health sub district	
CAMH policy and legislation	1. Adequacy of CAMH laws and policies; 2. Consciousness of CAMH laws and policies	Mental health policy exists	3	1	0	4
		CAMH policy exists	3	0	0	4
		Adequate CAMH related laws	1	0	0	1
Financing for CAMH	1. Government financing 2. Donor financing	Inadequate public funding	3	1	3	7
CAMH service delivery	1. Service adequacy 2. Integration	Availability of adequate services	0	0	0	0
		Inadequate services	3	1	1	5
		Services integrated	0	0	0	0
		Complementary services exist	1	0	0	1
CAMH health workforce	1. Numbers of CAMH workforce 2. Training	Adequate Numbers of CAMH work force	0	0	0	0
		Inadequate numbers of CAMH workforce	3	1	2	6
		Training of CAMH personnel present	3	1	0	4
CAMH medicines	Medicines sufficiency	CAMH medicines included on essential drug list for Uganda (EDLU)	3	1	2	6
		Adequacy of available medicines for CAMH	2	1	2	5
CAMH health information management and research	1. HMIS competence 2. Mental health reporting	HMIS adequacy	3	0	2	5
		Reports on CAMH exist	0	0	0	0
		CAMH research exists	1	0	0	0

### CAMH laws and policies

Two predominant themes emerged under CAMH laws and policies: (1) adequacy of CAMH laws and policies; and (2) awareness of CAMH laws and policies.

#### Adequacy of CAMH laws and policies

Existing global agreements and national laws supportive of CAMH include the United Nations Convention on the Rights of the Child; the 1995 Constitution of the Republic of Uganda; The Mental health Treatment Act, 1964; and the Children Act 1996. These were considered as sufficient for the promotion of CAMH, both by administrative and clinical managers at the central level. The Ministry of Health representative highlighted that physical, mental and social dimensions of child health were represented in these laws, which obliged the country to ensure child protection. It was reported at the national level that CAMH policy guidelines had been recently drafted to complement the national mental health policy.

#### Awareness of CAMH policies

However, even if CAMH policy was acknowledged at the national level, the managers at the district level were not aware of it. At the district level it was also noted that the national mental health policies were inadequate.

“We have the draft policy on mental health…But it is not widely distributed. The one I have is old, from around 2002. The policies are not adequate and they do not have direct focus on children and adolescent mental health.”*—*District Health Official

### CAMH financing

Government and donor financing emerged as themes under financing. Insufficient public financing for CAMH services was emphasized by all informants. This stemmed from an experienced underfunding of all health services leading to the district managers using scarce primary health care (PHC) resources for CAMH. Neither was any donor funding for CAMH noted.

“We do not have any particular development partners supporting mental health”—District health official

However, in-kind support in the form of collaborations, workforce development and refurbishment of infrastructure was acknowledged at a small scale.

### CAMH service delivery

Service Adequacy and Integration were emergent themes identified under CAMH service delivery.

#### Service adequacy

Inadequate quality and quantity of CAMH services was cited by all managers at national and district level, and the absence of CAMH or other mental health services at lower health centers (HC II and HC III) was mentioned as a contributor to this status. Only tertiary level services were acknowledged, as the excerpt illustrates:

“What you can call reasonable services are at the National Referral Hospital and Mulago National Hospital…from general hospital below, (there is) nothing”*—*Ministry of Health official

This was confirmed by district level managers who mentioned that lower level CAMH services were primarily dealing with epilepsy:

“At HC II and III the only condition they handle is epilepsy.”*—*District health official

Inpatient services were considered to be sufficient at the national level, but were noted as a particular challenge at the district level. Managers at all levels agreed that the range of CAMH services being provided is limited; and psycho-social services were quoted only in the national referral hospital. There were no community outreaches or promotional campaigns as noted by the managers in the following quotations:

“I have never seen any [promotional] campaigns in this district. Not even in Kampala.”*—*Health sub district manager

“The other modalities of treatment—behavioral therapy and so on they are not really [provided]”—Ministry of Health official

#### Integration

Integration of mental health and CAMH into other health sector services was also described as lacking. HIV services were specifically mentioned as an example where integration is absent:

“…Many of them (People living with HIV/AIDS) get some mental health problems. Some of them get obvious psychosis, depression, suicide attempts…but they (HIV services) are not capturing them.”*—*Ministry of Health official

Linking CAMH to child and adolescent services outside the health sector was also mentioned to be lacking; including outreaches to schools, communities, traditional healers and collaboration with the police and social welfare departments. Action from police and social welfare was cited in relation to forensic CAMH services:

“*…*they wait for children to commit crimes; that is when they appear to take the children to remand homes.”*—*Ministry of Health official

Attempts by the district health office to address substance and alcohol abuse in schools were curtailed by a lack of funding, as mentioned:

“We did some outreaches in schools mainly on drug abuse. Mainly in primary schools. Due to funding we are not consistent.”*—*District health official

The need for integration of CAMH into the education services was mentioned as a deterrent to school dropout and misunderstanding of children’s behavior.

“If you talk about epilepsy…the stigma that is associated with it means that these people cannot attend school and sometimes they drop out of school. If you can educate the student and the teachers I think that can help to improve mental health. ADHD for example, when they [teachers] see someone squirming and fidgeting they punish them—yet they can be helped.”—Official at national referral hospital

### CAMH workforce

Insufficient CAMH workforce and training emerged as the main themes under CAMH workforce. The insufficiency of the health workforce is widely cited. The numbers are few, the placement is inappropriate and the civil service staffing norms do not support recruitment and placement of mental health workers at lower level facilities, as illustrated by the informant from the Ministry of Health:

“It is not adequate. At the moment we have only three child and adolescent psychiatrists in the country.”*—*Official at national referral hospital

“The human resources have been very lacking”*—*Ministry of Health official

However, one lower level manager felt that staffing at his clinic was adequate:

“At this health center I think they are adequate—if we have the psychiatric nurse, and the other nurses, and the medical officers in my opinion that should really be adequate.”*—*Health sub district manager

To strengthen the existing workforce small scale training initiatives were ongoing at the national level, in collaboration with foreign donors. However at the district level no CAMH in-service training had been conducted.

“We are not doing in service training on mental health. Almost all our staff have not been sensitized on mental health and it is one of our missing links.”*—*District health official

### CAMH medicines

Medicine sufficiency was the only theme identified under CAMH medicines. Managers at all levels agreed that the Essential Drug List for Uganda (EDLU) included sufficient CAMH medicines and that availability of medicines at lower levels health facilities was adequate. *“The medicine supplies have improved of recent.”*—District health official

Nevertheless, managers noted that where trained staff were present, medicines were procured; thus medicines availability was dependent on staffing. They specifically noted that specialty medicines were not included on the EDLU due to lack of specialized staff to administer them.

“…but we only put at those levels where there is a service [provider] because we could not justify … special medicines when we know that the prescribers will not know how to use them… Availability is limited by human resources.”*—Ministry of Health official*

### CAMH health information management and research

Competency of health management information system (HMIS) and sufficiency of mental health reporting are the themes that emerged under this domain of the CAMH health system.

The HMIS is generally considered adequate for planning at the national level. However at the district level health managers consider the HMIS inadequate to support their planning and implementation of CAMH, partly because it does not disaggregate data into child and adolescent ages. However, there were divergent views on this lumping of CAMH data, exemplified by these two excerpts from national and district officials:

“It captures just a line “childhood mental disorders … for the time being we are contented to … lump … childhood mental disorders.”*—*Ministry of Health official

“It is inadequate…they do not break it down into specific diagnostic conditions…we do not capture 5–17 years. It does not tell us much and we do not have information on what occurs in the community.”*—*District health official

The lack of periodic reports on mental health in general and CAMH in particular was noted. The only opportunity to report on the national mental health status is in the Annual Health Sector Performance Report, in which a paragraph on mental health can be published. Ongoing CAMH research was noted by two national level managers,

“[in] The Annual Health Sector Performance report we have a page. Unfortunately [there is] no paragraph on child mental health—there are very small numbers who are being seen.”*—*Ministry of Health official

## Discussion

This assessment set out to explore the strengths and weaknesses of the health system for CAMH at the national and district levels in Uganda, with a focus on Mbale district, from a management perspective. We undertook a qualitative assessment of health managers’ perspective on policies and laws, financing, partnerships and collaboration, service delivery, health workforce, CAMH medicines and health information systems.

Contrary to previous research [13], in this study health managers report that the laws of Uganda promote CAMH. Even if the Mental Health Treatment Act of 1964 does not mention CAMH, subsequent laws, notably the 1995 Constitution of Uganda, which is the supreme law [24] and subordinate laws including the Children Act 1996 provide for protection of the child, implicitly including CAMH. These laws were perceived by the health managers as sufficient for supporting CAMH. The difference between our findings and previous research might be attributed to the fact that unlike previous studies, we did not undertake an analytic review of the laws and policies.

Similarly, unlike studies done before the CAMH policy was drafted, we found a perception among health managers that Uganda’s health policies promote CAMH. The draft National Mental Health Policy mentions the specific need for a CAMH policy, a new draft of which is in place. This reflects a recent priority placed on CAMH. As noted elsewhere, policy is an important aspect of mental health service scale-up [25].

The recent prioritization may be responsible for the fact that managers at district and sub district levels are unaware of the draft CAMH policy. However, it is worth noting that sub-district managers were unaware too of the draft mental health policy which has been in place since 2000. This points to a lack of policy dissemination from national to lower levels and is consistent with previous research in Uganda and elsewhere that cites insufficient dissemination of mental health policies as a barrier to mental health policy implementation [26, 27]. Policy dissemination usually involves distribution of policy booklets, accompanied by dissemination workshops if resources are available [27]. Suggestions for strengthening policy dissemination and implementation cited in the literature include involvement of district-level managers in policy development processes; engagement of different sectors that are relevant to CAMH and commitment of sufficient technical and financial resources to ensure policies are disseminated and implemented [13, 26, 27]. Investigation into barriers to effective policy dissemination specific to Uganda is warranted.

Opportunities for promoting CAMH lie in other child and adolescent service sectors [28, 29]. This assessment however reveals no knowledge among health managers of integration of CAMH within and outside the health sector. The lack of referral linkages with traditional healers contributes to the gap between CAMH conditions and the health system, bearing in mind that traditional healers in Eastern Uganda manage a substantial burden of mental ill health in communities [30]. The feasibility of integrating CAMH into other child and adolescent services; police services; and engaging with traditional healers to improve CAMH referral could be further explored.

We found that according to health managers, CAMH services are provided mainly at national and regional levels with no community outreach. At the health service levels below the RRH, managers acknowledged that CAMH services are largely absent. This is in direct contrast to recommendations that services should be decentralized from referral hospitals and cities to the communities [28, 31, 32]. In addition to being highly centralized, CAMH services in Uganda were considered by managers to be largely psycho-pharmacologic in nature in spite of a wide body of evidence in favor of non-pharmacologic forms of therapy, including psychotherapy and behavioral therapy [28, 33, 34].

The centralized nature of services can be attributed to inadequate numbers and distribution of CAMH workforce. The inadequate workforce is however not limited only to lower levels but affects the national level as well. As noted by a manager at national level, only three child psychiatrists serve the entire country. The difference in opinion regarding workforce sufficiency between the lower level managers on the one hand; and district and national level managers on the other hand reflects relativism, where the lower level managers’ opinions might be shaped by their contextual understanding of the health system, which is driven by perceptions of their sub-district.

Insufficient workforce was believed by managers to have limited the range of CAMH services being offered. The unavailability of behavioral therapy even at the national referral hospital is attributed to a scarcity of clinical psychologists in the country. This scarcity is confirmed by surveys which estimated at most two psychologists working in the mental health sector per 10,000,000 Ugandans [9, 15] and points to the need to implement CAMH workforce development strategies e.g. task sharing to non-specialist staff in primary care settings [35, 36].

Workforce insufficiency impacts availability of medicines as well. As noted by multiple informants, the availability and sufficiency of CAMH medicines in lower level clinics depends on the presence of staff competent enough to procure and prescribe the required medicines. At the national level however, we found agreement that the CAMH medicines were available for commonly treated conditions, such as epilepsy.

Disagreement between national and lower level managers on HMIS sufficiency for service planning existed, with lower level managers believing that the HMIS is insufficient. Lower level managers particularly felt that the current inability of the HMIS to capture children and adolescents is a major gap. This finding is consistent with results from the WHO’s 2005 mapping of CAMH resources which suggested a disconnect between availability of epidemiological data and planning needs [6]. This disconnect is likely to impact the development of evidence-based CAMH policies and programs.

Overall, the results of this assessment complement the quantitative results in previous studies, including the 2005, 2011 and 2014 Mental Health Atlas reports from WHO, which found that mental health financing, service access, human resources and medicines were insufficient in Uganda [9, 12, 15]. This assessment highlights an improvement in mental health policy since the 2011 Mental Health Atlas report.

We recognize some limitations of this study. While these data come from all the health managers responsible for CAMH at national level, the opinions of district health managers in this research cannot be generalized to all districts. Additionally, the research did not include an analytic review of policies, laws and government documents to validate the managers’ opinions. Lastly, the position of the PI as a Ugandan medical doctor with extensive experience in national and district health systems could have inhibited the lower level managers and biased their depictions of the health system. The PIs position is also a strength, however, as it might have influenced the interviewees to provide more candid information than it would have been if the interviewer were from outside the field.

## Conclusion

There are divergent perceptions among CAMH managers in Uganda on availability and adequacy of CAMH policy and laws; and the sufficiency of health information systems. However, managers agree that most components of the CAMH system in Uganda are weak, characterized by poor financing, inadequate quality and quantity of services, sparse human resources, and non-integration within health and non-health sectors. More effective dissemination of national policies to address the disparate policy opinions; CAMH workforce development to address the human resource gap; and increased integration of CAMH into primary health care and other sectors are suggestions for improving the availability and quality of CAMH services.

## Abbreviations

CAMH: Child and Adolescent Mental Health
DALY: disability adjusted life years
DHO: District Health Officer
EDLU: essential drug list of Uganda
HC: Health Center
HIV: human immunodeficiency virus
HMIS: Health Management Information System
HSD: Health Sub District
PHC: primary health care
PI: principal investigator
RRH: Regional Referral Hospital
WHO: World Health Organization
